# Superlative Artistic Abilities in a Patient With Post-traumatic Brain Injury

**DOI:** 10.7759/cureus.16697

**Published:** 2021-07-28

**Authors:** Anuj Kunadia, Shelby Aughtman, Michael Hoffmann, Fabian Rossi

**Affiliations:** 1 Internal Medicine, University of Central Florida College of Medicine, Orlando, USA; 2 Internal Medicine and Neurology, University of Central Florida College of Medicine, Orlando, USA; 3 Clinical Neurophysiology Laboratory, Orlando Veterans Affairs Medical Center, Orlando, USA

**Keywords:** diaschisis, superlative abilities post-traumatic brain injury, tbi compensatory ability, neurology, post traumatic brain injury

## Abstract

This case describes a patient who exhibits newfound superlative abilities in painting, music, philosophy, culinary, and performing arts after a traumatic brain injury (TBI) involving the frontal and temporal lobes. Such a dramatic change in de novo artistic behavior after brain injury is rare but has been reported in other patients with frontotemporal dementia, as well as other neurological diseases. Previous studies have shown that mild frontal cortical dysfunction likely plays a role in facilitating creative endeavors and that artistic circuitry is distributed throughout the brain. The neuronal reorganization which occurs after injuries enhances synapse formation and neural plasticity, which may contribute to the acceleration of artistic output after brain injury. This is likely an underdiagnosed phenomenon and a deeper understanding is required to allow clinicians to more effectively recognize and nurture newfound creativity in the setting of brain damage.

## Introduction

Long-term consequences of focal injuries within the cortex may manifest as increased compensatory functions from non-injured parts of the brain with the learning of behavioral strategies which compensate for the initial deficit [1, 2]. This ability of the brain to change its neuronal circuitry in response to injury is part of a phenomenon known as diaschisis [3-5]. Previous cases of this phenomenon have been documented, with a small number noting a significant growth of artistic abilities following traumatic brain injury (TBI), but these have largely been limited to the acquisition of new skills involving a single artistic medium or, if multiple, were due to non-traumatic causes [6, 7]. This case describes a patient who exhibits newfound superlative abilities in painting, music, philosophy, oratorship, culinary, and performing arts after a TBI involving the frontal and temporal lobes.

## Case presentation

A 40-year-old male with a history of post-traumatic stress disorder (PTSD) presented with a severe TBI after an all-terrain vehicle (ATV) accident, after which he lost consciousness. He received inpatient therapy and rehabilitation for difficulty with reading, walking, memory impairments, anosmia, and visual changes including photosensitivity, diplopia, and sudden, episodic vision loss. He reported “electric” sensations as though he is “hooked up to a generator” with “a live wire going down the arms and legs”. Additionally, the patient reported having seizures, falls, and occasionally dropping items from his hands. The patient also experienced mood swings and outbursts, though no manic symptoms were mentioned in the history. Physical examination was significant for recall of zero out of five words after five minutes, anosmia, dysgeusia, right-sided deafness, absent ankle jerk reflex, impaired light touch, and vibration sense in upper extremities to wrists and lower extremities to the ankles. He also had hypoesthesia on the left leg and left lateral calf. Tandem gait and Romberg’s test were both abnormal. Other physical examination findings were within normal limits.

**Figure 1:**
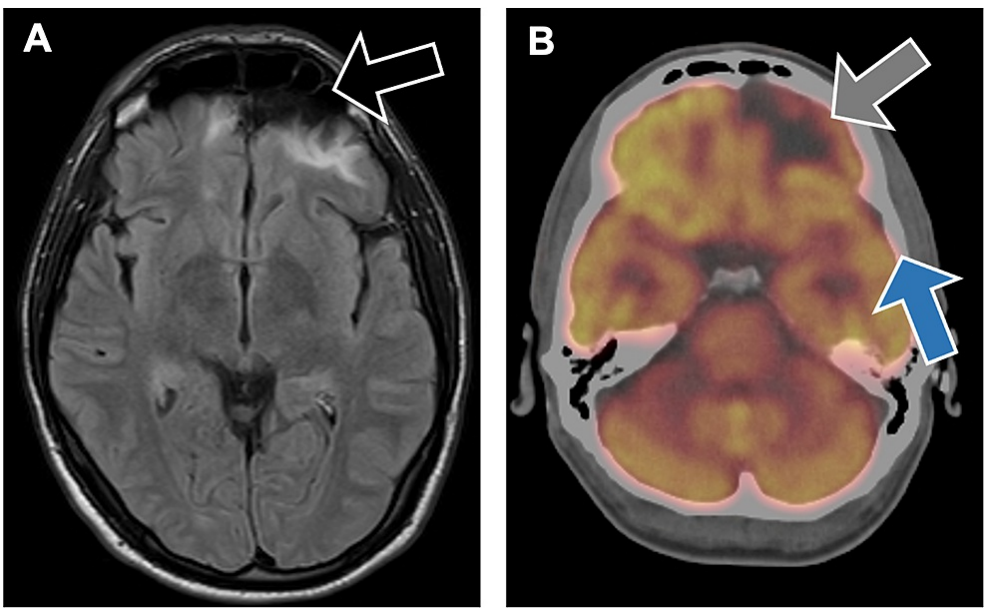
A. MRI brain scan revealing encephalomalacia of both inferior frontal lobes (black arrow) and left anterior temporal lobes related to the patient's traumatic brain injury. B. Positron emission tomography brain scan demonstrating photopenia in the left frontal lobe (gray arrow) and to a lesser extent in the left temporal (blue arrow) and right medial frontal lobe.

Imaging was performed during a follow-up visit three years after the incident and confirmed his existing diagnosis of encephalomalacia in both inferior frontal lobes and the left anterior temporal lobe secondary to his injury (Figure 1). The imaging findings are similar to those taken at the time of his TBI three years prior. The patient’s treatment plan included valproate to help control his seizures and serve as a mood stabilizer, as well as a trial of a non-invasive vagal stimulator for his refractory migraines. The patient was adherent to the treatment plan.

**Figure 2:**
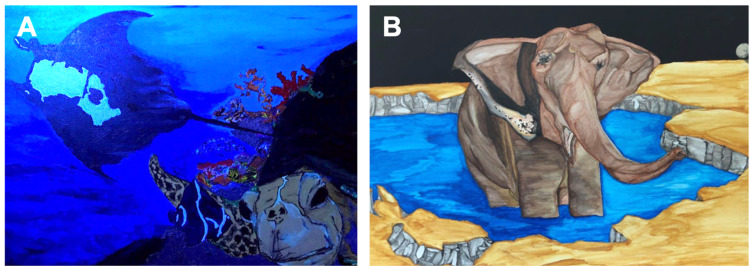
Paintings demonstrating the patient’s superior artistic ability more than three years after TBI involving the left anterior frontal lobe and left anterior temporal lobe. Illustrations depict a stingray and turtle (A) and an elephant (B). The patient reports having no prior experience with art prior to his TBI. TBI: Traumatic brain injury.

Starting a few months after his TBI and continuously developing throughout the three years following his initial presentation, the patient has continued to recover and has demonstrated heightened interest and new-onset ability in many artistic media. These include, but are not limited to, painting, graphic arts, music, and singing. At his most recent visit, his artistic output had continued to improve, with him revealing eight new remarkably outstanding pictures, based on the physician’s judgment. For reference, the patient reports having no prior practice in painting or other areas of art in which he developed a marked interest after his TBI. Figure 2 depicts two examples of the patient's artwork. Additionally, he noted improved dexterity, and his language and vocabulary had likewise markedly improved compared to those prior to his TBI. Along with these advances, he reported increased skills in composing and playing music, writing poetry, and rapping. In addition, he started a radio talk show and reported flourishing culinary skills through cooking remarkable meals (as reported by the patient and the patient’s wife), involving unique combinations of exotic ingredients. The patient reported becoming more philosophical and indulges in many metaphysical preoccupations, self-described as "intuition on steroids". Examples of the patient's artistic abilities are summarized in Table 1.

**Table 1:** Areas of increased interest in the patient after his TBI with examples of corresponding productive output. TBI: Traumatic brain injury.

Area of Interest	Example(s)
Illustrative Artistry	Paintings of deep-sea creatures and scenes, elephant and other animals, perceptual art
Performing Arts	Creation of radio show
Music	Creation of rap music and writing poetry
Culinary Arts	Preparation of exquisite cuisines
Oratorship	Increased vocabulary
Philosophy	Highly philosophical with increased interest in philosophical literature
Religion	Increased interest in religion

Cognitive screening, neuropsychiatric and behavioral testing were performed using psychometric scales depicted in Table 2. Taken together, these collectively demonstrated the presence of a frontotemporal syndrome, as evidenced by the Rascovsky and DAPHNE-6 criteria [8]. In addition, the subsyndrome, Gastaut-Geschwind syndrome (GGS), was evident in the context of relatively intact cognitive ability (Montreal Cognitive Assessment [MoCA] score 26/30). The patient’s Bear-Fedio inventory was positive for altered sexual interest, circumstantiality, interpersonal viscosity, sense of personal destiny, exaggerated philosophical concern, religiosity, and hypergraphia (conventionally 3 of 18 criteria are required to diagnose GGS [9, 10]. Of note, GGS has previously been associated with increased pan-artistic abilities, as seen in this patient. Frontotemporal syndrome was diagnosed using DAPHNE-6 criteria (positive for disinhibition, apathy, loss of empathy, perseverations, hyperorality, and neglect) [8]. The Frontal Behavioral Inventory (FBI) score and Frontal Systems Behavior Scale (FrSBe) scores were all in the abnormal range, the same for the executive function score.

**Table 2:** Behavioral neurological test scores. MoCA: Montreal Cognitive Assessment.

Test	Score	Interpretation
MoCA	26	Score ≥ 26 supports normal cognitive function
DAPHNE-6	4	Score ≥ 4 supports frontotemporal dementia
Frontal Behavioral Inventory	35	Score ≥ 27 supports frontotemporal syndrome
Bear and Fedio Inventory	7/18	Score ≥ 3/18 required to diagnose Gastaut-Geschwind syndrome
Frontal Systems Behavioral Evaluation - Apathy/Abulia; Disinhibition; Executive function; Total score	65; 74; 59; 69	T scores ≥ 60 are abnormal

## Discussion

Brain injury, whether in the form of TBI or ischemic stroke, results in neuroanatomical effects involving damage, recovery, and compensation of neural pathways in the brain. A cascade of events can be triggered at the primary site of damage, carrying devastating effects such as interrupted blood supply and cell death from glutamate-mediated excitotoxicity [11, 12]. After the initial injury, tissues may undergo swelling and inflammation, compromising the integrity of sites distant from the primary focus of damage [12]. These swollen and inflamed tissues can cause deficits that may vary from mild and transient to debilitating and permanent [3]. While triggering such detrimental events described above, brain injuries may also induce a cascade of events involved in growth, allowing intact neurons to survive, self-repair, and build new connections [13]. Increased expression of growth-promoting genes, along with cortical reorganization, neurogenesis, axonal sprouting, dendritic plasticity, and angiogenesis [14] facilitates an environment for optimal neuronal growth [15].

In one study of primates, researchers observed an expansion of the hand area of the ventral premotor cortex following a lesion of the area in the ipsilateral hemisphere [16]. These changes were associated with increased axonal sprouting [17] and an overall improvement in motor behavior. After injuries, there is typically increased dendritic growth in the hemisphere contralateral to the lesion, as well as a significant increase in axonal growth [18] and a number of synapses [19], a pattern observed within many different species, including humans. The effect of TBI on the emergence of *de novo* artistic behavior following traumatic injury or disease, while rare, has been reported in other patients with frontotemporal dementia, epilepsy, subarachnoid hemorrhage, and Parkinson’s disease. There is likely a role for mild frontal cortical dysfunction resulting in the production of certain behavioral and cognitive characteristics that may facilitate the creation of art.

A review of two world-renowned artists, Braque and Kokoschka, both of whom suffered TBIs during World War I, demonstrated full recoveries in their artistic abilities. Each artist continued producing exceptional art that remained highly regarded and influential. The review suggests a widely distributed and diffuse neural control by the brain in the creation of art, symbolic cognition, and abstract thinking. This contrasts with other areas of the brain, like those involved in language, which are relatively more confined. One possible evolutionary explanation for this is that art supplements language by facilitating the sharing of emotions, experiences, ideas, thoughts, and symbols of social identity. For this reason, diffuse artistic circuitry in the brain is thought to have promoted bonding among the early groups of Homo sapiens in efforts to survive harsh environmental conditions with scarce food sources. This could be one of the underlying reasons for how art became a form of communication, supplementing language [20]. By granting such evolutionary advantages, artistic ability is not localized to one place in the brain but rather distributed throughout. As described earlier, a significant reorganization of neuronal circuitry occurs following a TBI, with increased synaptic connections and axonal growth. It may, then, stand to reason that such a significant change in the neuronal organization would impact artistic ability and creativity. This patient’s presentation, recovery, and compensation suggest extensive neural reorganization through diaschisis, especially in the right cerebral hemisphere, contralateral to the TBI. These changes in his neurons may have played a key role in the drastic acceleration of his artistic capabilities.

## Conclusions

Such an increase in artistic abilities is likely an underdiagnosed phenomenon, creating an opportunity to further understand the neural foundation of creative thought, as well as the manifested changes which occur after brain injury. The case presented is unique and to the authors’ knowledge, pan-artistic abilities of such magnitude have not been reported in the literature. As clinicians, a deeper understanding of these changes can help us more effectively recognize and cultivate newfound creativity and talent in the setting of TBI, stroke, and other neurological diseases, helping patients and their families find solace and optimism during difficult times.
